# A 10‐year prediagnostic follow‐up study shows that serum RNA signals are highly dynamic in lung carcinogenesis

**DOI:** 10.1002/1878-0261.12620

**Published:** 2020-01-10

**Authors:** Sinan Uğur Umu, Hilde Langseth, Andreas Keller, Eckart Meese, Åslaug Helland, Robert Lyle, Trine B. Rounge

**Affiliations:** ^1^ Department of Research Cancer Registry of Norway Oslo Norway; ^2^ Department of Clinical Bioinformatics Saarland University Saarbrücken Germany; ^3^ Department of Neurology and Neurological Sciences School of Medicine Stanford University CA USA; ^4^ Department of Human Genetics Saarland University Homburg Saar Germany; ^5^ Department of Oncology Oslo University Hospital Norway; ^6^ Institute for Cancer Research Oslo University Hospital Norway; ^7^ Institute of Clinical Medicine University of Oslo Norway; ^8^ Department of Medical Genetics Oslo University Hospital and University of Oslo Norway; ^9^ Faculty of Mathematics and Natural Sciences PharmaTox Strategic Research Initiative School of Pharmacy University of Oslo Norway; ^10^ Department of Informatics University of Oslo Norway

**Keywords:** lung cancer, NSCLC, prediagnostic serum, RNA dynamics, RNA‐seq, SCLC

## Abstract

The majority of lung cancer (LC) patients are diagnosed at a late stage, and survival is poor. Circulating RNA molecules are known to have a role in cancer; however, their involvement before diagnosis remains an open question. In this study, we investigated circulating RNA dynamics in prediagnostic LC samples, focusing on smokers, to identify if and when disease‐related signals can be detected in serum. We sequenced small RNAs in 542 serum LC samples donated up to 10 years before diagnosis and 519 matched cancer‐free controls coming from 905 individuals in the Janus Serum Bank. This sample size provided sufficient statistical power to independently analyze time to diagnosis, stage, and histology. The results showed dynamic changes in differentially expressed circulating RNAs specific to LC histology and stage. The greatest number of differentially expressed RNAs was identified around 7 years before diagnosis for early‐stage LC and 1–4 years prior to diagnosis for locally advanced and advanced‐stage LC, regardless of LC histology. Furthermore, NSCLC and SCLC histologies have distinct prediagnostic signals. The majority of differentially expressed RNAs were associated with cancer‐related pathways. The dynamic RNA signals pinpointed different phases of tumor development over time. Stage‐specific RNA profiles may be associated with tumor aggressiveness. Our results improve the molecular understanding of carcinogenesis. They indicate substantial opportunity for screening and improved treatment and will guide further research on early detection of LC. However, the dynamic nature of the RNA signals also suggests challenges for prediagnostic biomarker discovery.

## Graphical abstract

We investigated circulating RNA dynamics in prediagnostic lung cancer (LC) samples, focusing on smokers, to identify if and when disease‐related signals can be detected in serum. The results showed dynamic changes in differentially expressed circulating RNAs specific to LC histology and stage. The dynamic RNA signals identified different phases of tumor development over time and were associated with cancer‐related pathways.

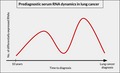

**Abbreviations**
ADCs: adenocarcinomas
BDg: blood donor group
CT: computed tomography
JSB: Janus Serum Bank
LC: lung cancer
lncRNAs: long noncoding RNAs
MAPK: MAPK signaling
miRNA: microRNA
miscRNAs: miscellaneous RNAs
NSCLC: non‐small‐cell lung cancer
PI3K‐Akt: PI3K‐Akt signaling
piRNAs: piwi‐interacting RNAs
RAS: Ras signaling
RNA‐seq: RNA sequencing
SCLC: small‐cell lung cancer
snoRNAs: small nucleolar RNAs
tRFs: tRNA‐derived fragments

## 1 Introduction

Lung cancer (LC) is the leading cause of cancer deaths worldwide (National Lung Screening Trial Research Team *et al.*, 2011b; Urman and Hosgood, 2016). There are two major histologies of LC: non‐small‐cell lung cancer (NSCLC), representing approximately 85% of cases with adenocarcinomas (ADCs) and squamous cell carcinomas as the main histological subtypes (Chen *et al.*, 2014; Gridelli *et al.*, 2015; Herbst *et al.*, 2008), and small‐cell lung cancer (SCLC), constituting about 15% of cases (Gazdar *et al.*, 2017; Herbst *et al.*, 2008). Despite improvements in therapies, LC survival is poor. Survival increases with early‐stage diagnosis (Brustugun *et al.*, 2018; National Lung Screening Trial Research Team *et al.*, 2011b), but only 25% of patients are diagnosed at this stage (National Lung Screening Trial Research Team *et al.*, 2011b). Screening methods such as low‐dose computed tomography (CT) can be effective for early detection (National Lung Screening Trial Research Team *et al.*, 2011a) and reduce LC mortality in high‐risk groups (WCLC, 2018). However, it has a high false‐positive rate (Gopal *et al.*, 2010), and annual CT scans cause harmful radiation exposure (Bach *et al.*, 2012). High‐risk groups also need to be better defined to increase screening effectiveness (Osarogiagbon *et al.*, 2019). Therefore, there is a pressing need to understand the molecular changes occurring prior to disease to be able to develop noninvasive biomarkers of LC.

Bodily fluids, including serum, contain a rich repertoire of circulating RNA molecules (Fehlmann *et al.*, 2018; Fritz *et al.*, 2016; Umu *et al.*, 2018) that originate from nonmalignant and malignant cells. RNAs play a central role in cellular processes (Fritz *et al.*, 2016) and in tumor metastasis (Steenbeek *et al.*, 2018) and have been proposed as cancer biomarkers (Inamura and Ishikawa, 2016; Zaporozhchenko *et al.*, 2018). Among them, microRNAs (miRNAs) have been extensively studied as LC biomarkers (Chen *et al.*, 2012; Inamura and Ishikawa, 2016; Keller *et al.*, 2011; Leidinger, Keller, and Meese, 2012; Wang *et al.*, 2016). Other RNA types, such as Piwi‐interacting RNAs (piRNAs), isomiRs, tRNAs, small nucleolar RNAs (snoRNAs), mRNAs, and long noncoding RNAs (lncRNAs), are also found in bodily fluids (Fritz *et al.*, 2016; Kim, Abdelmohsen, Mustapic, Kapogiannis, and Gorospe, 2017; Umu *et al.*, 2018), but are not as well studied, and their functions in circulation are mostly unknown.

The biomarker promise of miRNAs has remained largely unfulfilled (Cho, 2011; Wang *et al.*, 2016; Witwer, 2015). For example, from 32 studies, 143 breast cancer‐related miRNA biomarkers were reported. Of these, 100 were replicated only once, 25 had discordant expression direction, and the remainder had low expression fold change (Witwer, 2015). One of the reasons why very few RNA biomarkers are in clinical use is because of the lack of reproducibility across studies due to differences between patient groups, sample materials, and methodologies (Keller and Meese, 2016; Wang *et al.*, 2016; Zaporozhchenko *et al.*, 2018). Moreover, traits like age, sex, smoking, body mass, and physical activity are associated with RNA expression and will confound the discovery and use of RNAs as cancer biomarkers (Keller and Meese, 2016; Rounge *et al.*, 2018).

Another reason why RNAs are not extensively used as LC biomarkers is our limited understanding of prediagnostic molecular dynamics. Disease progression causes temporal variation in RNA expression driven by cellular mechanisms such as genetic and epigenetic changes, angiogenesis, cellular energy consumptions, immune activation, avoidance and growth, metastasis, and cell death (Gutschner and Diederichs, 2012; Peng and Croce, 2016; Pichler and Calin, 2015). As a consequence, prediagnostic RNA levels might be histology‐specific, highly dynamic, and nonlinear (Holden *et al.*, 2017; Lund *et al.*, 2016) as opposed to gradual. Such dynamic patterns require large sample sizes, a long prediagnostic time window, and long‐term follow‐up to capture. Understanding circulating RNA dynamics will improve our knowledge of the molecular basis of cancer, which in turn can improve cancer diagnosis, treatment, and prevention. However, no previous LC studies have investigated prediagnostic RNA expression dynamics in depth.

**Figure 1:**
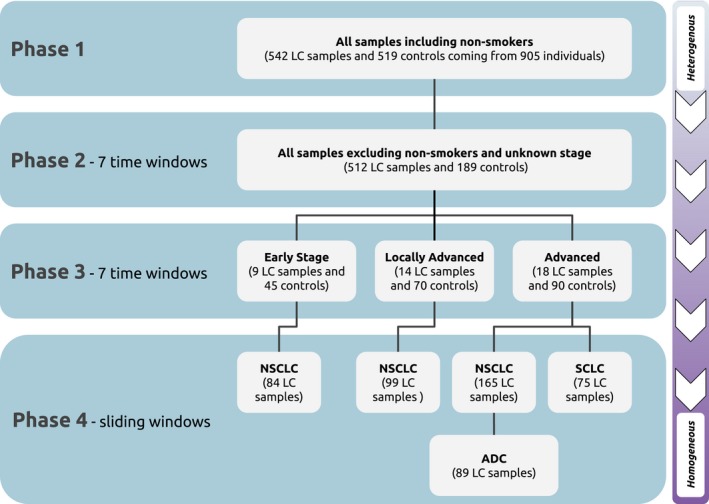
Each phase adds another aspect to our design which confirms time, stage, and histology dependence of prediagnostic signals. This chart is summarizing the different phases of the analyses with sample sizes and methodologies. In phase 1, we used an all‐vs‐all approach that contains more heterogeneity in the analysis. This resulted in a weak signal (Fig. S4). In phases 2 and 3, all stages were represented with identical number of samples in each time window to balance statistical power and contribution of each stage into the signals. In phase 4, we used a sliding window approach. Stages and histologies were analyzed separately to increase homogeneity in the analysis. For more information about included and excluded sample numbers, see Fig. S2

In this study, we measured RNA levels in 542 serum samples from LC patients collected up to 10 years before their diagnosis and 519 frequency‐matched cancer‐free controls from healthy donors (Fig. 1). The samples were classified according to histology, stage, and time to diagnosis (Fig. S1). We identified highly dynamic prediagnostic RNA levels and enriched functional pathways that clearly signal cancer progression many years before the diagnosis. Our focus is to investigate the dynamic nature of prediagnostic RNA levels rather than discovering LC biomarkers.

## 2 Materials and methods

### 2.1 Study design and participants

The Janus Serum Bank (JSB) cohort is a population‐based cancer research biobank containing serum samples from 318 628 Norwegians (Hjerkind *et al.*, 2017; Langseth *et al.*, 2017). For inclusion of samples to the JanusRNA study, we linked the JSB cohort to the Cancer Registry of Norway (Larsen *et al.*, 2009). We sequenced 542 prediagnostic serum samples from 391 LC patients donated up to 10 years before their diagnosis (Fig. S1). As controls, we sequenced 519 serum samples from 518 donors who were cancer‐free (except from nonmelanoma skin cancer) at least 10 years after sample collection. LC samples and controls were frequency‐matched on sex, age at donation, and blood donor group (BDg). BDg is a technical cofounder combining the effect of storage time and sample treatment at donation (Rounge *et al.*, 2018). LC samples were stratified based on matching criteria. We randomly selected controls such that the case–control ratio was the same for each stratum.

The JanusRNA study contains a rich set of clinical and epidemiological data enabling analyses of specific subsets of LC. Clinical data at the time of diagnosis were classified as NSCLC, SCLC, and ‘others’ with less defined or multiple histologies. The LC samples were classified using the TNM system into four stages: early (verified localized stage—stage I), locally advanced (clinically or pathologically verified regional stage—stages II and III), advanced or metastatic (clinically or pathologically verified distant stage—stage IV), and unknown (unknown stage, or cases with insufficient information) (Cancer Registry of Norway, 2018) (Table 1 and Fig. S1). LC stage at the time of diagnosis does not necessarily reflect the tumor stage at the time of sample donation. We used stage with the assumption that rapidly growing tumors are more likely diagnosed at a late stage and slower growing tumors can be diagnosed at an early stage.

**Table 1:** Summary of sample and patient characteristics.

	Control	Stage			
		Early (localized)	Locally advanced (regional)	Advanced (distant)	Unknown
Histology					
NSCLC	–	84	101	171	11
SCLC	–	9	35	76	4
Others	–	10	5	32	4
Sex					
Male	350	78	104	180	12
Female	169	25	37	99	7
Age at donation, years					
Mean (SD)	49.9 (11.2)	54.3 (7.33)	55.0 (9.04)	53.4 (8.26)	51.8 (6.53)
Smoking					
Yes^a^/No	189/330	102/1	139/2	271/8	19/0
Prediagnostic sampling time, years					
Mean (SD)	–	5.52 (2.81)	5.63 (2.78)	5.89 (2.66)	6.75 (2.18)
Age at diagnosis, years					
Mean (SD)	–	59.8 (7.67)	60.6 (8.84)	59.3 (8.35)	58.6 (6.05)
Total samples	519	103	141	279	19
Total individuals	905				

a Including former and current smokers.

Smoking is categorized as current, former, or never smokers (Hjerkind *et al.*, 2017). Almost all cases with smoking status available were current or former smokers. For phase 1, all cases and controls were included regardless of smoking status. For phases 2, 3, and 4 (see Section 2.4 below), 11 LC samples that reported to be never smokers or had missing data were excluded (Fig. S2). We included only cases and controls that were former or current smokers in these phases, resulting in a total of 531 LC samples and 189 controls.

### 2.2 Serum RNA profiling

RNA was extracted from 400 µL serum using phenol–chloroform and miRNeasy Serum/Plasma kit (Qiagen, Valencia, CA, USA). Libraries were prepared with the NEBNext Small RNA kit (NEB, Ipswich, MA, USA) and sequenced on a HiSeq 2500 (Illumina, San Diego, CA, USA) as previously described (Umu *et al.*, 2018). RNA profiles from the JSB healthy donor samples show high RNA diversity, including sncRNAs and also fragments of longer RNAs (lncRNAs and mRNAs) (Rounge *et al.*, 2015; Umu *et al.*, 2018).

### 2.3 Bioinformatics, case–control matching, and differential expression analyses

We compiled a comprehensive annotation set from miRBase (v22) (Kozomara and Griffiths‐Jones, 2014) for miRNAs, piRBase for piRNAs (Zhang *et al.*, 2014), and GENCODE (Harrow *et al.*, 2012) for other RNAs. This dataset included 10 circulating RNA classes, miRNA, miRNA hairpin, isomiR, piRNA, tRNA, tRF, snoRNA, miscRNA, lncRNA, and mRNA. For the RNA sequencing (RNA‐seq) data, we filtered out RNAs with fewer than 5 reads in less than 20% of the samples. We used MINTmap for tRF (Loher *et al.*, 2017) and SeqBuster for isomiR profiling (Pantano *et al.*, 2010). Other bioinformatic details are available in our previous study (Umu *et al.*, 2018). The *optmatch* r package (http://github.com/markmfredrickson/optmatch) identified LC samples and matched controls for analyses. This tool enabled us to select optimal sets of control samples when there are enough controls to select from (Fig. S3 shows an age matching example).

The deseq2 r package (v1.18.1) (Love *et al.*, 2014) was used for the differential expression analyses with default settings. We performed KEGG pathway analyses for mRNAs, miRNA targets, and isomiR targets. We used R function *kegga* from the *limma* package. The miRNA and isomiR targets were extracted from mirdb (v5.0) predictions (Wong and Wang, 2015) (score cutoff > 60).

### 2.4 Analysis design

We analyzed RNA expression for all samples (phase 1), dependent on prediagnostic time (phase 2), stage (phase 3), and histology (phase 4) (Fig. 1 and Fig. S2). Prediagnostic time was divided into seven discrete ~ 17‐month‐long time intervals in phases 2 and 3. The intervals were optimized for statistical power and resolution of time prior to diagnosis. To make the time windows comparable with respect to statistical power, each window has the same number of LC samples and controls. They also have similar proportions of stages and histology when possible.

For phase 1, RNA levels were analyzed using age, sex, smoking, and BDg as confounders in the DESeq2 model. For this phase only, we assigned smoking status ‘unknown’ to samples with missing smoking information since DESeq2 does not accept samples with missing data (NAs). For the remaining phases, all included cases and controls were former or current smokers. For phase 2, we selected 27 LC samples matched on stages and 135 matched controls per time window.

For phase 3, we selected 9 LC samples and 45 matched controls for early LC, 14 LC samples and 70 matched controls for locally advanced LC, and 18 LC samples and 90 matched controls for advanced‐stage LC. For replication analysis, we randomly resampled phase 3 samples 20 times without replacement for each time window to bootstrap the variance of the signal. In each iteration, we randomly selected new LC samples and rematched new controls. To further test the robustness of the signals, we chose 2‐year time intervals instead of 17 months. This changes sample selection in each time frame substantially.

For phase 4, we used a sliding window approach to analyze prediagnostic RNA expression dynamics dependent on stage and histology. The sliding window approach is agnostic to critical time windows since it creates a continuous RNA signal trajectory. We chose 17‐month‐long windows and 2.5‐month‐long step size to provide the smallest possible window size while maintaining statistical power. Resampling for each window is analogous to the replication in phase 3. We did not have enough samples to analyze early and locally advanced SCLC and ADC separately (Fig. 1).

### 2.5 Ethics approval and consent

This study was approved by the Norwegian Regional Committee for medical and health research ethics (REC no: 2016/1290) and was based on a broad consent from participants in the Janus cohort. The work has been carried out in compliance with the standards set by the Declaration of Helsinki.

## 3 Results

### 3.1 Small differences in RNA levels between LC cases and controls

By using all LC samples (*n* = 542) vs controls (*n* = 519) (Fig. 1), maximizing statistical power and also LC sample heterogeneity, we identified 88 differentially expressed RNAs (Fig. S4). The majority of these were tRNAs (43), followed by tRNA‐derived fragments (tRFs) (23), although some of these were likely overlapping or duplicated genes. The maximum effect size was low (−0.85 log_2_FC, *TMED2*), suggesting a weak overall signal. We did not detect significant enrichment of known pathways.

### 3.2 Prediagnostic RNA dynamics unveil strong time‐dependent signals

**Figure 2:**
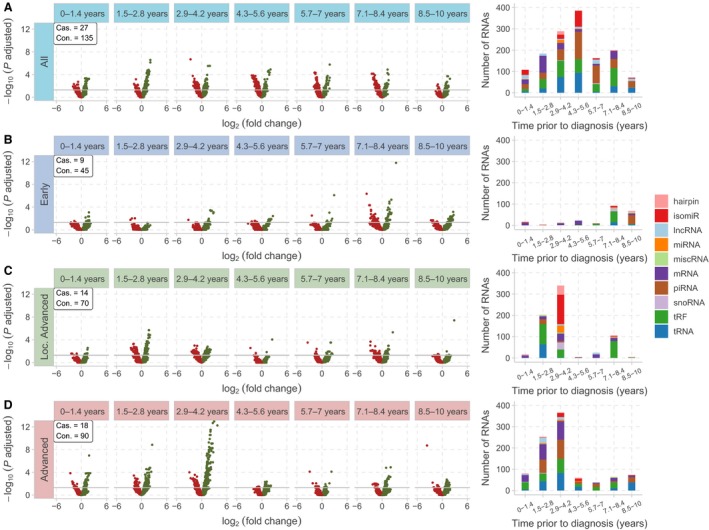
Prediagnostic RNA dynamics in patients with early, locally advanced, and advanced LC show stage‐ and time‐dependent signals. The volcano plots show the differential expression analyses for each time period of phases 2 and 3. The bar plots on the right side summarize the classes of differentially expressed RNAs. The gray lines on the volcano plots show the significance cutoff (*P*‐adjusted < 0.05), and each dot represents a different RNA (green, upregulated; red, downregulated), while the *x*‐axes show the effect sizes and *y*‐axes show the significance. (A) By combining the samples from all three stages (phase 2), we detected a strong peak at the interval 4.3–5.6 years. There are also relatively weaker signals in other intervals. (B) The early‐stage LC differential expression analysis results show two peaks in the time periods 7.1–8.4 and 8.5–10 years. We used 9 LC samples and 45 matched controls per volcano plot for this stage. (C) The locally advanced‐stage results have the strongest signal in the time periods 1.5–2.8 and 2.9–4.2 years. We utilized 14 LC samples and 70 matched controls for this stage. (D) The advanced‐stage results show two peaks in the time periods 1.5–2.8 and 2.9–4.2 years. Another small peak is at the time period 0–1.4 years. We utilized 18 cases and 90 matched controls for this stage.

Next, by separating LC samples according to prediagnostic time (still with high LC sample heterogeneity due to stage and histology), we identified 1400 differentially expressed RNAs in 7 time intervals with 27 LC samples and 135 controls in each interval (Fig. 1). The highest represented RNA types were piRNAs (387), tRFs (319), tRNAs (255), mRNAs (189), and isomiRs (130). We detected differentially expressed RNAs in every time interval with a gradually increasing numbers of RNAs approaching a peak at 5 years, followed by a steady decline until diagnosis (Fig. 2A). A total of 289 RNAs were detected in more than one time interval. For example, tRF‐20‐I8W47W1R, tRF‐21‐I8W47W1R0, tRF‐22‐I8W47W1RN, piR‐hsa‐12790, and piR‐hsa‐2106 were detected in 6 time intervals spanning approximately 9 years. tRF‐21‐I8W47W1R0 also had the strongest effect size, −3.71 log_2_FC (Table S1).

There are 84 significantly enriched pathways in total in these time windows, with the highest number at 3‐4 years prior to diagnosis. Cancer‐related pathways, such as MAPK, RAS*,* and *Pathways in cancer,* were among the most significantly enriched (Fig. S5 and Table S2). We identified enriched pathways 8–10 years before diagnosis including *Endocytosis*, *Wnt signaling*, and *Adherens junction pathway,* important in cell‐to‐cell communication*.* This time period also contained cancer‐related pathways like *Adrenergic signaling* and *Renal cell carcinoma.*

We confirmed the robustness of dynamic RNA signals in this phase by selecting 2‐year time windows. This substantially changes sample selection in each time window, but the results showed that the dynamic signal is robust (Fig. S6, ‘All’ panels).

### 3.3 Prediagnostic RNA dynamics in patients with early, locally advanced, and advanced LC show stage‐specific signals

We next separated LC samples in each window by stage to reduce heterogeneity of LC. For early stage LC, we identified 229 RNAs, using 9 LC samples and 45 controls in each interval (Fig. 1). The highest represented RNA types were tRFs (61), piRNAs (46), and mRNAs (49). The strongest signals were observed in two time intervals spanning 7–10 years prior to diagnosis; however, these intervals did not share any RNAs. Four RNAs were detected in more than one time interval of early‐stage analysis. Among these, tRF‐21‐I8W47W1R0 showed the strongest effect size, −3.71 log_2_FC, and was downregulated in two intervals (Fig. 2B and Table S1).

There were 12 significantly enriched pathways for early‐stage LC time intervals, and 10 of these were significant 7–10 years before diagnosis. *Axon guidance, Cell adhesion molecules, FoxO signaling*, PI3K‐Akt, and *Transcriptional misregulation in cancer* were among the most significant pathways. The signal 8–10 years before diagnosis also included *Endocytosis* and *Transcriptional misregulation* pathways.

For locally advanced stage LC, we identified 699 RNAs, using 14 LC samples and 70 controls in each interval. The most represented RNA types were tRFs (214), isomiRs (143), and mRNAs (94). The strongest differential expression signal was detected between 3 and 4 years. Forty‐six RNAs were detected in more than one time interval. *RAB21* mRNA produced the strongest effect size (log_2_FC −3.43), significantly downregulated 6–7 years before diagnosis (Fig. 2C and Table S1).

We detected 116 significantly enriched pathways for locally advanced LC. Almost all pathways, 112, were enriched in the time frame 3–4 years before diagnosis. The most significant pathways included *Axon guidance,* MAPK*, Pathways in cancer, mTOR signaling, ErbB signaling,* RAS*,* PI3K‐Akt, and *p53 signaling pathway*.

For advanced stage LC, we identified 936 RNAs, using 18 LC samples and 90 controls in each interval. The highest represented RNA types were mRNAs (219), piRNAs (211), tRNAs (199), and tRFs (193). The strongest signals were observed in the time periods 1–3 and 3–4 years before diagnosis, and these intervals shared 104 RNAs. A total of 205 RNAs were found in more than one interval. An isomiR (of hsa‐miR‐486‐3p) had the largest effect size and was significantly upregulated 3–4 years before diagnosis (Fig. 2D and Table S1).

We found 101 enriched pathways for advanced‐stage LC, 47 between 1 and 3 years, and 45 between 3 and 4 years. The most significant pathways were MAPK, *Axon guidance, Proteoglycans in cancer,* RAS*, ErbB signaling, Focal adhesion*, and *p53 signaling pathway*.

We assessed consistency of the LC signal and identified 236 differentially expressed RNAs in at least two stages at any time interval. Twenty‐seven of them were detected in all three stages, consisting mostly of tRFs (10) and mRNAs (8). A similar trend was observed between locally advanced and advanced stages for 1–4 years before diagnosis. A total of 112 RNAs were shared in this time interval, consisting mostly of tRNAs (36), tRFs (27), and mRNAs (23), and also 66 pathways. Among these pathways, we identified *NSCLC pathway, SCLC pathway, Pathways in cancer, Proteoglycans in cancer, Choline metabolism in cancer, Central carbon metabolism in cancer, p53 signaling,* MAPK*, mTOR signaling,* PI3K‐Akt, etc. A complete list of the enriched pathways and their significance is in the supplementary material (Fig. S5 and Table S2).

The replication using bootstrapping (Fig. S7) and 2‐year time intervals confirmed the robustness of dynamic and stage‐specific signals (Fig. S6, stage panels). However, we also identified minor variance in the bootstrapping results. Early stage showed overall lower variance, and the strongest signal around 7 years was consistent. Locally advanced and advanced stages produced some variance of the signals. The strongest signals were consistent for both stages, while we observed a signal around 5 years before diagnosis for advanced stage.

### 3.4 Prediagnostic RNA dynamics in NSCLC and SCLC by stage reveal histology‐specific signals

**Figure 3:**
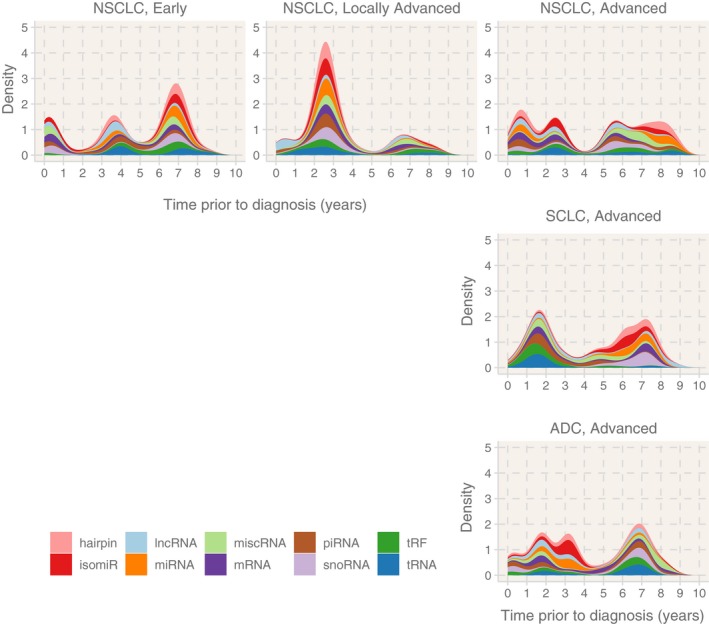
Prediagnostic RNA dynamics in NSCLC and SCLC by stage reveal histology‐specific signals. Each panel shows the RNA signal prior to diagnosis for all stages and histologies, identified with sliding window analyses (phase 4). Early and locally advanced SCLC and ADC histologies did not have enough samples (missing panels). The colors of the density plots represent different RNA classes. For example, the signal around 2.5 years of the locally advanced NSCLC displays differential expression of isomiRs (red), miRNA (orange), piRNA (brown), and tRNA (blue).

Lastly, we further reduced LC sample heterogeneity by including histology information. For early NSCLC, we identified two strong peaks around 4 and 7 years before diagnosis using a sliding window approach (Fig. 1); however, the composition of these peaks was different (Fig. 3). The peak around 7 years consisted mostly of isomiRs, tRFs, and miRNAs, whereas the peak around 4 years consisted mostly of piRNAs, tRFs, isomiRs, and miRNAs. For locally advanced NSCLC, we identified a strong peak around 2.5 years before diagnosis that consisted mostly of isomiRs, mRNAs, piRNAs, tRFs, and miRNAs. Another peak was detected around 7 years before diagnosis that consisted mostly of mRNAs, tRFs, isomiRs, and piRNAs (Fig. 3).

For advanced NSCLC, we detected two signals spanning years 0–3 and years 5–9 before diagnosis. These signals were similar in RNA composition, consisting mostly of tRFs, piRNAs, mRNAs, and isomiRs. However, the year 5–9 signal had more differentially expressed miscellaneous RNAs (miscRNAs) (Fig. 3). For advanced SCLC, we identified similar signal dynamics as advanced NSCLC. Two signals covered years 0–3 and years 4–8 before diagnosis. The year 0–3 signal contained mostly tRFs, tRNAs, piRNAs, and mRNAs, while the year 4–8 signal contained mostly isomiRs, mRNAs, and miRNAs (Fig. 3). miRNA‐hairpin structures were also detected in the year 4–8 signal, which suggested a strong miRNA‐related RNA differential expression. For advanced ADC, we also detected parallel signals as advanced‐NSCLC and advanced‐SCLC results. We found signals between years 0 and 4 containing mostly isomiRs, tRFs, and piRNAs, and years between 5 and 9 containing mostly tRFs, tRNAs, and piRNAs (Fig. 3).

## 4 Discussion

Our results clearly showed the dynamic nature of serum RNA signals up to 10 years before LC diagnosis. To the best of our knowledge, our study is the largest available to date with up to 10 years of follow‐up time investigating the major RNA classes in serum. This dataset has enabled us to investigate in depth dynamic changes in circulating RNA expression and enriched pathways with time, stage, and histology.

It is known that RNA expression levels are dysregulated at the time of a cancer diagnosis. However, there are very few studies investigating prediagnostic blood samples from cancer patients focusing on potential biomarkers. Keller *et al.,* based on a much smaller sample size from the same cohort and measuring miRNAs with array technology, found the strongest LC signal close to diagnosis. However, this study lacked information on stage, histology, and controls within the same cohort (Keller *et al.*, 2011). Other studies have shown the dynamics of protein coding mRNA levels in prediagnostic breast cancer samples, but the emphasis was on statistical methods (Holden *et al.*, 2017; Lund *et al.*, 2016). In phase 1, we used all available samples without taking time, stage, and histology into account. This analysis identified a few differentially expressed RNAs with reasonable FCs, and no enriched pathways. Taking prediagnostic time into account (phase 2) substantially increased the number of differentially expressed RNAs indicating a strong time‐to‐diagnosis dependency. The dynamic prediagnostic RNA signals that we see probably indicate the timing of the hallmarks of cancer (Hanahan and Weinberg, 2000) and periods of carcinogenesis, dormancy, and regression (Endo and Inoue, 2019; Massion and Carbone, 2003; Weis and Cheresh, 2013). Supporting this interpretation, the cancer‐related pathways derived from the dynamic RNA signals imply cancer hallmarks. More homogeneous LC sample selection by including stage (phase 3) and histology (phase 4) further increased the sensitivity and specificity of the prediagnostic signals. This indicates that regulation of specific pathways differs with histologies and stages. The clinical implications of this are that it may be possible to detect cancer early with a noninvasive screening method and improve patient survival. In addition, potential biomarkers may also help in choosing the best treatment options since the signals are specific to stage and histology which can indicate tumor aggressiveness.

The stage‐dependent pathway analyses (of phase 3) suggest that the functional signals were mostly related to cell‐to‐cell communication (e.g., *Endocytosis*) and cancer dormancy (e.g., *TGF‐beta signaling*) in early‐stage LC and cell proliferation in advanced LC stage (e.g. *EGFR*) (Fig. S5 and Table S2). We found specific enrichment of signaling pathways like EGFR, MAPK, RAS, PI3K‐Akt, and p53 signaling. This is striking since (a) most of the identified pathways were cancer‐related (Table 2), (b) the pathways were identified based on RNAs from blood where only a small fraction may originate from tumor tissue or tumor microenvironment, (c) there were clear cancer signals at multiple time points up to 10 years prior to diagnosis, and (d) some enriched pathways suggest transition between stages.

**Table 2:** Top 10 significantly enriched pathways in patients with early, locally advanced, and advanced‐stage LC based on phase 3. A detailed list of all significantly enriched pathways is in the supplementary material.

Pathway	Stage (prediagnostic time)	Adjusted *P*‐values
Axon guidance	Early (1.4–2.8)	2.83e‐3
	Locally advanced (2.8–4.2)	2.69e‐10
	Advanced (1.4–2.8), (2.8–4.2)	6.03e‐4, 6.62e‐7
MAPK signaling pathway	Locally advanced (2.8–4.2)	4.46e‐10
	Advanced (1.4–2.8), (2.8–4.2)	8.4e‐6, 6.62e‐7
Pathways in cancer	Locally advanced (2.8–4.2)	5.57e‐8
	Advanced (1.4–2.8), (2.8–4.2)	6.14e‐3, 6.16e‐3
Endocytosis	Early (8.4–10)	0.03
	Locally advanced (2.8–4.2)	1.90e‐7
	Advanced (1.4–2.8), (2.8–4.2)	0.0379, 6.16e‐3
Neurotrophin signaling pathway	Locally advanced (2.8–4.2)	1.17e‐6
	Advanced (1.4–2.8), (2.8–4.2)	0.0011, 0.018
HPV infection	Locally advanced (2.8–4.2)	1.27e‐6
	Advanced (2.8–4.2)	0.012
Ubiquitin mediated proteolysis	Locally advanced (2.8–4.2)	2.57e‐6
	Advanced (1.4–2.8)	0.013
mTOR signaling pathway	Locally advanced (2.8–4.2)	2.67e‐6
	Advanced (1.4–2.8)	8.3e‐4
ErbB signaling pathway	Locally advanced (2.8–4.2)	7.03e‐6
	Advanced (1.4–2.8), (2.8–4.2), (4.2–5.6)	0.29, 0.0012 9.94e‐5
Ras signaling pathway	Locally advanced (2.8–4.2)	7.03e‐6
	Advanced (1.4–2.8), (2.8–4.2)	0.013, 7.97e‐5

In early stage LC, the enriched pathways at 7–10 years before diagnosis included PI3K‐Akt signaling. Cancer cells secrete factors that inhibit PI3K‐Akt during serum deprivation (Jo *et al.*, 2008). We found that this pathway combined with *TGF‐beta signaling,* previously linked to dormancy (Klein, 2011; Weis and Cheresh, 2013), suggests an early phase of LC carcinogenesis. Many tRFs and piRNAs were also differentially expressed around 7 years prior to diagnosis; however, their roles are unknown.

In locally advanced stage LC, we identified pathways mostly at 3–4 years before diagnosis. The stage‐specific pathways included *VEGF signaling* and *TNF signaling*. VEGF is related to angiogenesis (Herbst *et al.*, 2008), and tumor cells secrete VEGF to ensure adequate blood supply (Gridelli *et al.*, 2015). TNF regulates cell proliferation in LC (Shang *et al.*, 2017), and it is an important therapeutic target (Ray *et al.*, 2010). There was a strong RNA signal around 7 years before diagnosis of locally advanced LC consisting of tRFs. They might point to an important event in LC progression even if their functional roles are unknown.

In advanced‐stage LC, the predominant signal identified 1–5 years prior to diagnosis was similar to the above‐mentioned locally advanced signal. However, *Hedgehog* and *GnRH* signaling pathways were specific to advanced LC. The Hedgehog pathway has an essential role in cell proliferation, survival, and differentiation, and aberrant regulation was linked to cancer (Yao *et al.*, 2018), including LC (Yuan *et al.*, 2007). It is regulated by various factors, including miRNAs and lncRNAs (Yao *et al.*, 2018). GnRH signaling is linked to LC progression, and GnRH agonists have strong antimetastatic, antiproliferative, and anti‐angiogenic activity (Lu *et al.*, 2015). Therefore, enrichment of these pathways may suggest strong metastatic activity.

Our results clearly showed that RNA signaling differs with staging at diagnosis even though the samples were collected prior to diagnosis. This indicates that stage at diagnosis may be used as a proxy for aggressiveness of tumor development. Early‐stage LC diagnosis may indicate slower progression, while locally advanced‐LC and advanced‐stage‐LC diagnosis may have a faster cancer progression. Thus, the prediagnostic RNA signal may indicate different disease trajectories. The bootstrapping and modifying the time intervals showed consistent RNA signals (Figs S6 and S7). The variation observed is likely a result of heterogeneous LC samples in this phase since we combined all histologies, suggesting that the histologies have a major effect on prediagnostic signals.

The fixed time interval (phase 3) or sliding window (phase 4) approaches select different samples for analysis but confirm the highly dynamic signals. All stage‐ and histology‐specific analyses showed at least two critical time windows (peaks) where LC differs from controls, and these were usually followed by time periods with no detectable signals. Peaks and troughs might potentially indicate tumor progression and dormancy.

The phase 4 results also contained RNA molecules that can be linked to early and advanced carcinogenesis that are specific to histologies (Fig. 3). For example, tRF‐21‐I8W47W1R0 was strongly downregulated (−4.05 log_2_FC) around 7 years before diagnosis in early‐NSCLC samples and 2 years prior to diagnosis in locally advanced‐ and advanced‐NSCLC samples. Another notable example is hsa‐miR‐483‐5p, which was previously found to promote metastasis of ADC (Song *et al.*, 2014). It was differentially expressed in advanced ADC around 7 years prior to diagnosis. Lastly, hsa‐miR‐184, also identified in phase 1, was upregulated in advanced NSCLC around 2 years before diagnosis and advanced ADC. hsa‐miR‐184 was previously proposed as a prognostic biomarker for SCLC (Zhou *et al.*, 2015), and it also downregulates MYC mRNA (Swier *et al.*, 2019).

Our study has multiple strengths. First, we selected case and matched control samples from a large cohort of serum samples with complete long‐term follow‐up, and we have detailed information on LC histology and stage. Second, extensive smoking information was available from health surveys enabling us to include only current or former smokers, thus reducing smoking‐related confounding from the LC signal. Third, we included samples at multiple time points prior to diagnosis. Fourth, the deep sequencing data contained all major RNA classes identified in serum. Finally, we included biological and technical confounders affecting circulating RNA levels in our dataset (Rounge *et al.*, 2018; Umu *et al.*, 2018).

However, there are some potential limitations. First, we lack completeness in the survey data, specifically from Red Cross blood donors (~ 10% of our samples). In analyses, when confounder information is critical (phases 2, 3, and 4), samples with missing information were excluded (Fig. 1). Second, we have to some degree included samples from the same individuals. However, since they hardly appeared at the same time windows, we considered the effect to be negligible. Third, we adjusted *P*‐values for multiple testing in each time window, but we did not take into account the overall number of tests. Fourth, some analyses were not done due to insufficient number of samples (Fig. 3), which may have also caused imperfect matching of controls for some analyses (Fig. S3). This can also partly explain some of the variance. Fifth, long‐term storage may degrade some unstable RNA molecules, but our previous study suggests that this effect is limited (Umu *et al.*, 2018). Finally, pathway analyses only included miRNA and isomiR predicted targets and mRNA fragments, since for other RNA classes, there are no functional predictions available.

## 5 Conclusion

This study clearly shows that LC signals can be detected in serum RNA up to 10 years prior to diagnosis. The highly dynamic signals are time‐to‐diagnosis‐, stage‐, and histology‐dependent and indicate disruption of cancer‐related pathways detectable in circulation. This is very promising for LC biomarker discovery and indicates a substantial opportunity for screening and improved treatment.

## Conflict of interest

The authors declare no conflict of interest.

## Author contributions

HL and TBR devised the project and gathered the samples. RL and TBR designed and coordinated laboratory work and sequencing. SUU performed analyses and drafted the manuscript. SUU, TBR, RL, and HL wrote the article in consultation with AK, ÅH, and EM. All authors provided critical feedback and helped to shape the article.

## Supporting information


**Fig. S1.** The distribution of LC case samples based on stage, histology, and prediagnostic time.
**Fig. S2.** Consort figure.
**Fig. S3.** Age of individuals.
**Fig. S4.** Phase 1 results and the volcano plot.
**Fig. S5.** Enriched pathways.
**Fig. S6.** Comparison of two different time intervals.
**Fig. S7.** Bootstrapping analysis for phase 3.

**Table S1.** RNAs over time and stage.

**Table S2.** Table of enriched pathways.

## Data Availability

The datasets generated and analyzed during the current study are not publicly available since individual privacy could be compromised, but are available from the corresponding author on reasonable request and with appropriate approvals.
