# Supplementary material for: A 10‐year prediagnostic follow‐up study shows that serum RNA signals are highly dynamic in lung carcinogenesis
Source: Mol Oncol. 2020 Jan 10;14(2):235–47. doi: 10.1002/1878-0261.12620 (PMC6998662; doi:10.1002/1878-0261.12620)
Supplement: Supplementary file 1 — Fig. S1. The distribution of LC case samples based on stage, histology, and prediagnostic time. Fig. S2. Consort figure. Fig. S3. Age of individuals. Fig. S4. Phase 1 results and the volcano plot. Fig. S5. Enriched pathways. Fig. S6. Comparison of two different time intervals. Fig. S7. Bootstrapping analysis for phase 3. [file MOL2-14-235-s001.docx]

Supplementary Information:

**Prediagnostic serum RNA levels are highly dynamic in lung cancer**

**Authors**

Sinan Uğur Umu^1,*^, Hilde Langseth^1^, Andreas Keller^2,3^, Eckart Meese^4^, Åslaug Helland^5,6,7^, Robert Lyle^8,9^ and Trine B. Rounge^1,10,*^

**
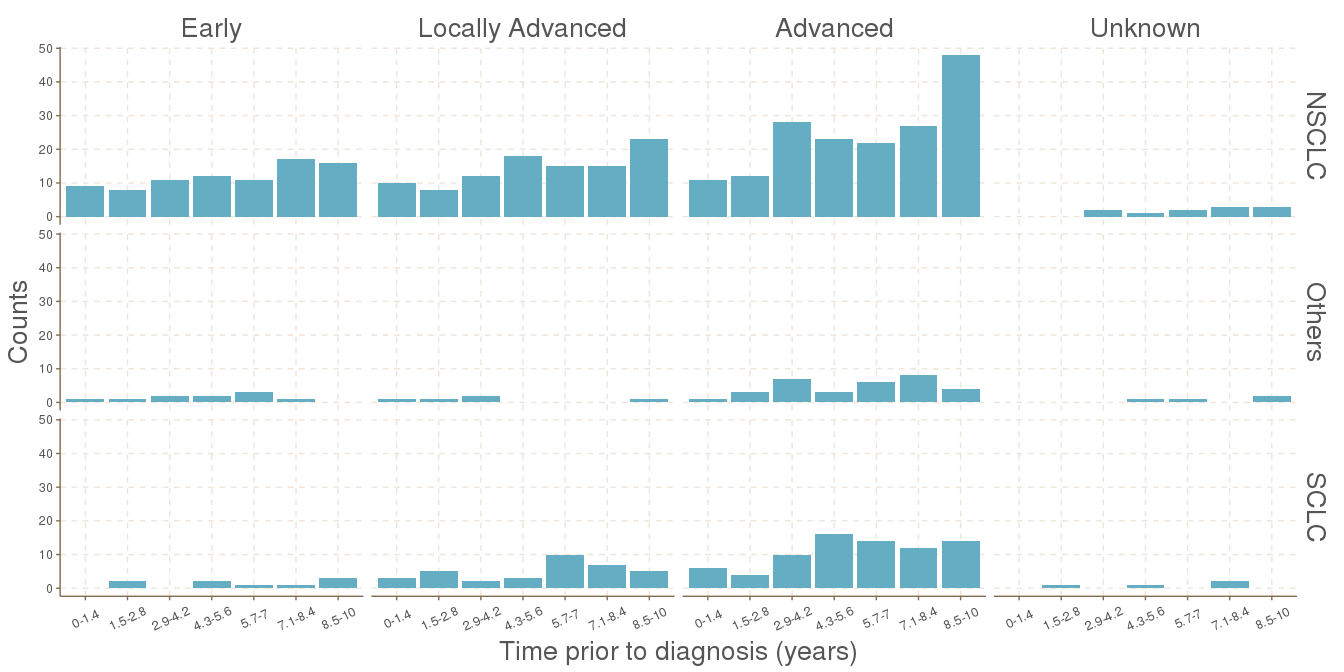
**

**Figure S1.** The distribution of LC case samples based on stage, histology and prediagnostic time. This plot shows that most of the samples are NSCLC advanced followed by NSCLC locally advanced. We do not have many samples with unknown stage and/or other histology.


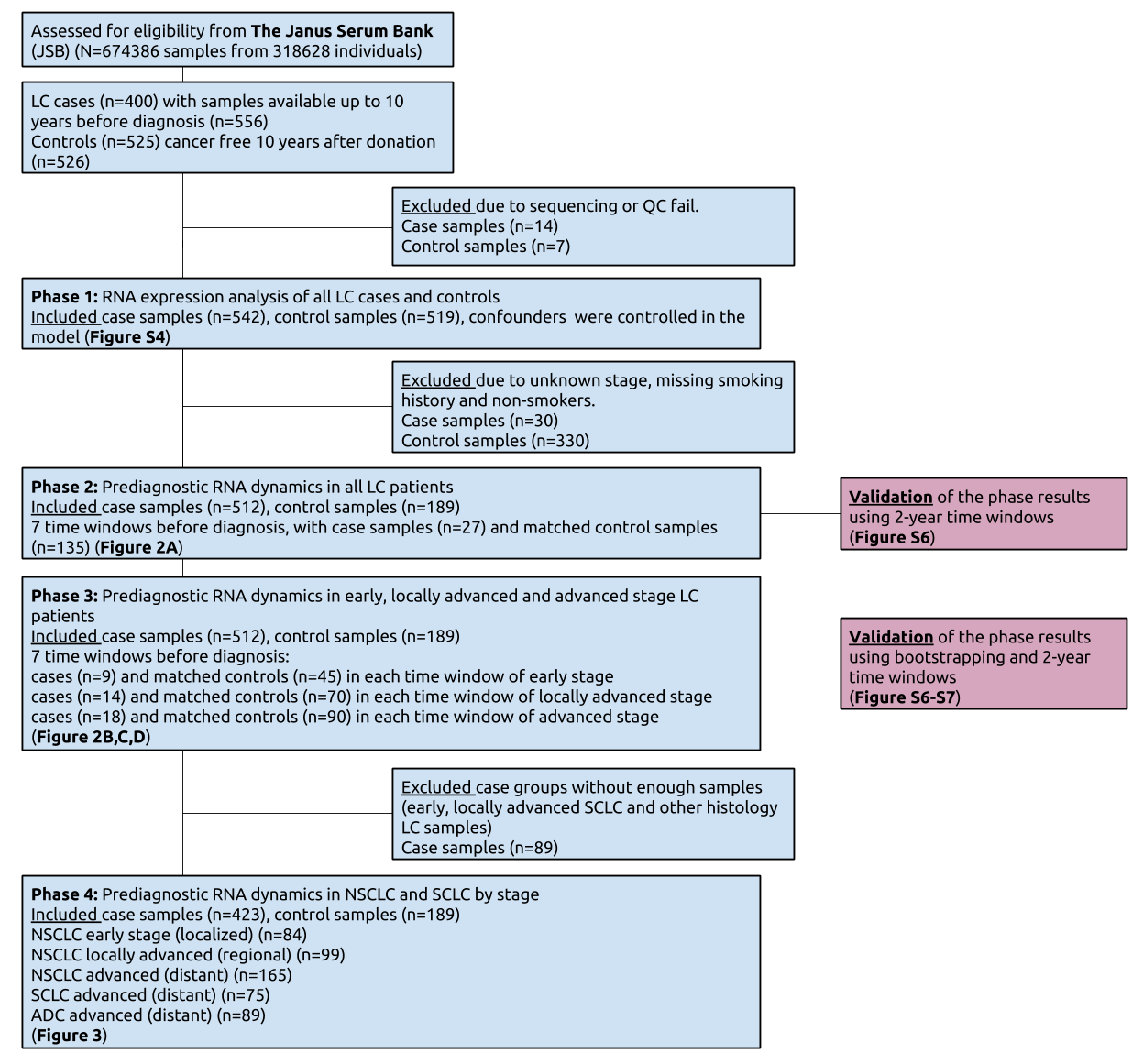


**Figure S2.** Flow-chart showing sample cases and sample controls included and excluded in the different phases of the analyses.


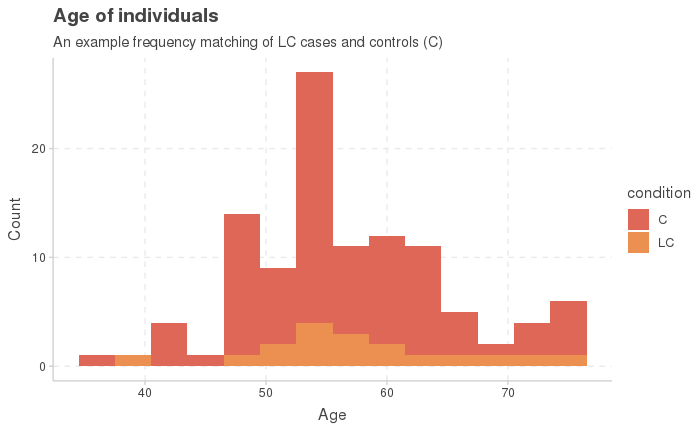


**Figure S3.** This histogram shows age distribution of individuals in two groups, LC cases (n=18) and controls (n=90), which displays an example frequency matching. There is no difference between these two groups (t-test *p*=0.57).


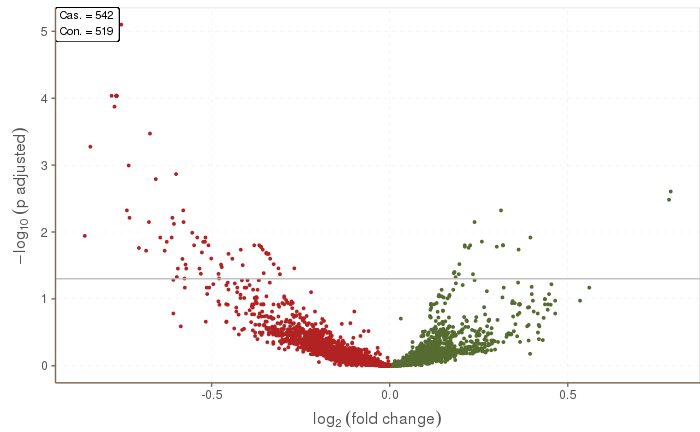
**Figure S4.** This volcano plot summarizes the differential expression analysis for all LC samples vs controls including non-smokers. As explained, we did not include, prediagnostic time, stage or histology into this analysis. DESeq2 model included blood donor group (BDg), age, sex and smoking as confonders. The grey line shows the significance threshold (*p*-adj. < 0.05). Red dots show the downregulated RNAs while green shows the upregulated ones. Only 88 RNAs were differentially expressed which showed no enriched pathways.


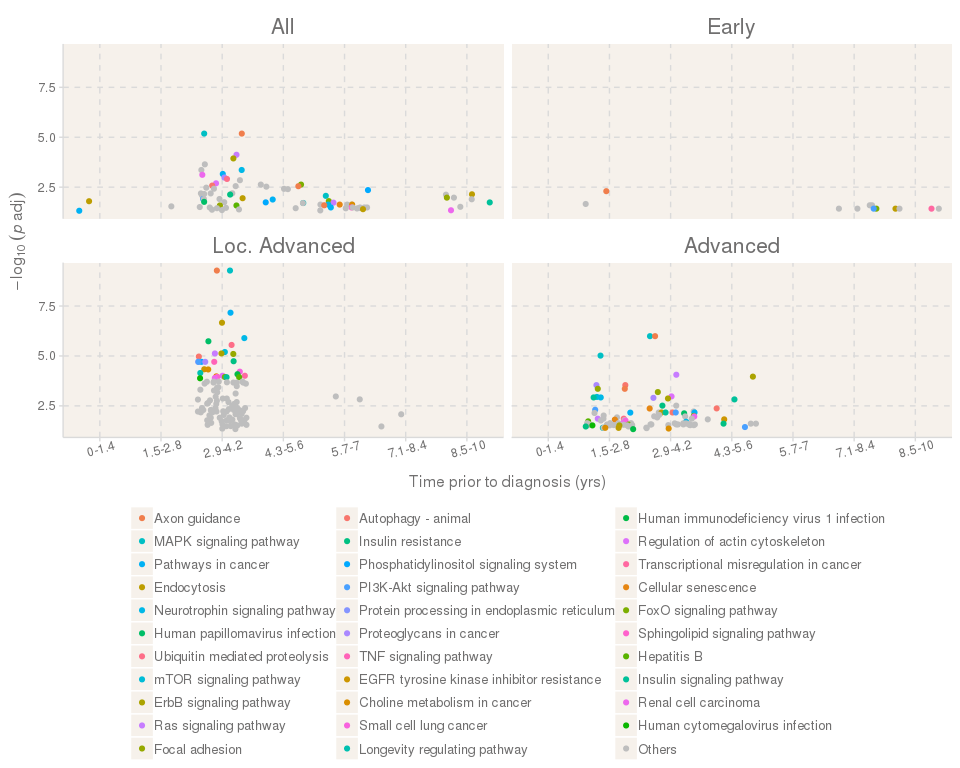


**Figure S5.** These four panels present the enriched KEGG pathways among prediagnostic samples. We used phase 2 and phase 3 results to enrich pathways. The x-axes show the prediagnostic time and the y-axes show the statistical significance. The legend below colors the top 32 most significantly enriched pathways that differentially expressed miRNAs (targets), mRNAs and isomiRs (targets) are involved. The grey points represent the rest of the significantly enriched pathways. The results reveal that many cancer-related pathways were enriched prior to diagnosis.


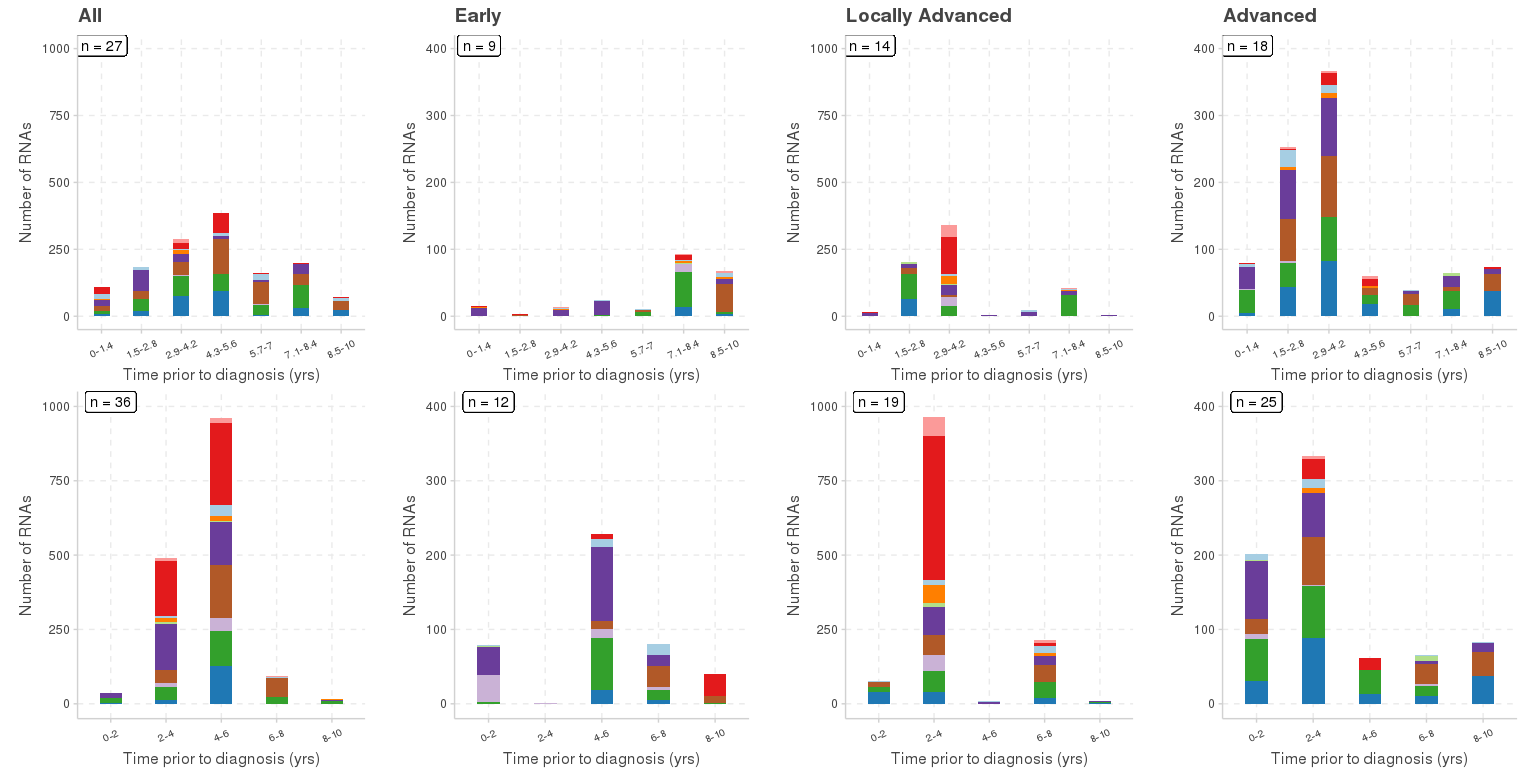


**Figure S6.** These plots show the comparisons between two different time intervals: the panels above show 17-months and the panels below show 2-years long time intervals. The results show similar trends. Differences in statistical power and sample heterogeneity can explain some of the variation (like in early stage). The total number of case samples in each window is visible at top.


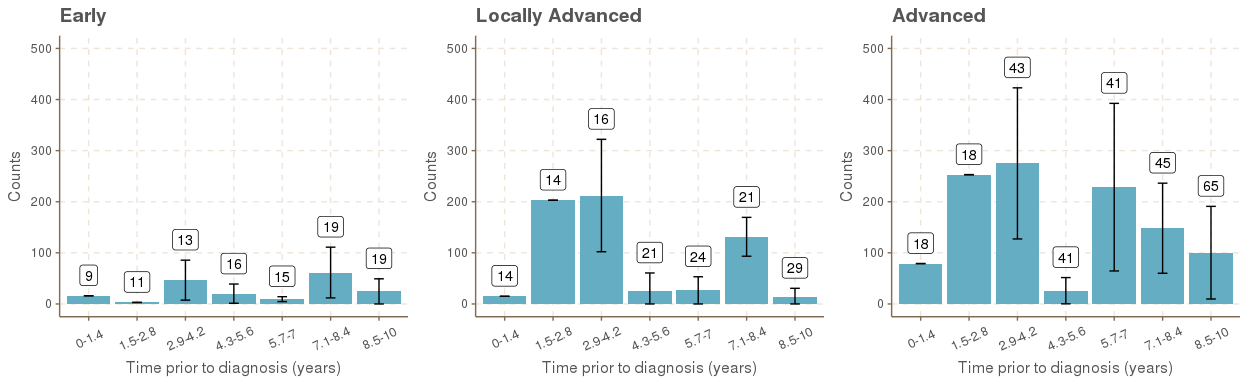


**Figure S7.** These plots show the bootstrapping results of phase 3 analyses. The signals identified are similar to the original (Fig. 2 barplots) but some variation was also observed. Each window was randomly subsampled 20 times. The labels show how many cases were in the reserve. The higher number means the more difference in subsamples. We did not observe a direct relationship between them. However, when the reserve was too small (e.g. early stage), the variance was also weak.
